# Atomoxetine‐Associated Bilateral Testicular Pain and Priapism in a Patient Taking Aripiprazole: A Case Report

**DOI:** 10.1155/crps/6645625

**Published:** 2026-07-17

**Authors:** Anoop Narahari, Kavita Kothari

**Affiliations:** ^1^ Department of Psychiatry, Baton Rouge Medical Center, Baton Rouge 70809, Louisiana, USA; ^2^ Department of Psychiatry, Atrium Health, Concord 28025, North Carolina, USA, atriumhealth.org

**Keywords:** aripiprazole, atomoxetine, case report, priapism, testicular pain

## Abstract

Priapism, a urologic emergency, is defined as a penile erection persisting longer than 4 h independent of sexual stimulation. While psychotropic medication–associated priapism is well documented, atomoxetine‐associated priapism remains extremely rare. Atomoxetine is a norepinephrine reuptake inhibitor used in the management of attention‐deficit/hyperactivity disorder (ADHD). We report the case of an Asian male in his late 20s with bipolar I disorder who had been treated with aripiprazole 7.5 mg daily for 6 months and developed sudden bilateral testicular pain, followed by a painful erection lasting more than 4 h, beginning 5 days after starting atomoxetine. He had no history of genitourinary trauma, malignancy, or concurrent medication or substance use. Clinical and laboratory evaluation were unremarkable, and detumescence occurred approximately 6 h after symptom onset without invasive intervention. Atomoxetine was discontinued the next day and no recurrence was reported at 4‐week follow‐up. Causality assessment using the Naranjo Adverse Drug Reaction Probability Scale yielded a score of 4 (possible) for atomoxetine. This case underscores the importance of counseling patients prescribed atomoxetine and other psychotropics about priapism and the need for urgent evaluation when erections persist for ≥4 h.

## 1. Introduction

Priapism is a urologic emergency because prolonged ischemic episodes can cause corporal hypoxia, fibrosis, and permanent erectile dysfunction, making timely recognition and prompt management essential [1, 2]. Erectile dysfunction is defined as the inability to attain and/or maintain a penile erection sufficient for satisfactory sexual performance. Priapism is classified into three subtypes: ischemic (low‐flow), non‐ischemic (high‐flow), and stuttering (recurrent ischemic) [1]. Detumescence is primarily mediated by sympathetic outflow via α1‐adrenergic receptors; therefore, medications with α1‐adrenergic antagonism can impair detumescence and predispose to ischemic priapism [2, 3]. Ischemic priapism is the most common form and is typically characterized by painful rigidity and dysregulation of arterial blood inflow and venous outflow. Risk factors include hematologic disorders (e.g., sickle cell disease and glucose‐6‐phosphate dehydrogenase deficiency), malignancies, perineal trauma, recreational drug use, and medications—most commonly psychotropics (e.g., trazodone and antipsychotics) and α1‐adrenergic antagonists [2, 4].

Atomoxetine is a selective norepinephrine reuptake inhibitor approved by the United States (U.S.) Food and Drug Administration (FDA) as a nonstimulant treatment for attention‐deficit/hyperactivity disorder (ADHD) [5]. Rare genitourinary adverse events, including priapism and testicular pain, are described in post‐marketing reports, FDA safety communications, and the U.S. prescribing information [5, 6]. Because ischemic priapism can lead to corporal fibrosis, erectile dysfunction, penile deformity, and need for surgical intervention when prolonged, early recognition, and patient education are essential [1, 2].

## 2. Case Report

A male in his late 20s of south‐Asian ethnicity with a psychiatric history of bipolar I disorder, well controlled on aripiprazole 7.5 mg daily for approximately 6 months without recent dose adjustments, was diagnosed with attention‐deficit hyperactivity disorder (ADHD). Atomoxetine was initiated at 40 mg daily, which is the standard recommended starting dose for adults with ADHD [5]. A medication history was obtained; aside from aripiprazole, he reported no other prescribed medications, over‐the‐counter (OTC) products, supplements, or erectogenic agents (e.g., phosphodiesterase‐5 inhibitors). Illicit drug use was denied.

Five days after initiating atomoxetine, he developed sudden bilateral testicular pain, followed by a painful penile erection lasting more than 4 h that was not associated with sexual activity. He had no prior history of priapism. His medical history was otherwise unremarkable, with no known sickle cell disease, hematologic malignancy, urogenital trauma or surgery, pelvic or perineal injury, or recent infections. He denied urinary symptoms, fever, penile discharge, or prior similar episodes. Concerned about priapism, he presented it to the emergency department for evaluation. On ED arrival, the vital signs were stable. Physical examination revealed mild bilateral testicular tenderness without swelling, erythema, or palpable masses. Genital examination demonstrated rigid penis, tender to palpation, and intact distal sensation. No inguinal lymphadenopathy or systemic abnormalities were noted.

Laboratory investigations, including complete blood count and basic metabolic panel, were within normal limits. Given the duration (≥4 h) and associated pain, the presentation was most consistent with suspected ischemic priapism; however, cavernosal blood gas analysis and penile Doppler ultrasonography were not obtained because detumescence began spontaneously during observation and the erection resolved within approximately 6 h of symptom onset.

The management consisted of observation without pharmacologic or procedural intervention. The erection resolved spontaneously approximately 6 h after symptom onset and did not require corporal aspiration, intracavernosal sympathomimetic injection (e. g., phenylephrine), or surgical intervention. Atomoxetine was discontinued the day after the emergency department visit (day 6 of therapy). Causality assessment using the Naranjo Adverse Drug Reaction Probability Scale yielded a score of 4 (possible) for atomoxetine (Table S1). At 4 weeks follow‐up, he reported complete resolution of testicular pain and no recurrence of priapism. No alternative ADHD medication was initiated, and there were no changes to his aripiprazole dose.

## 3. Discussion

This case describes acute bilateral testicular pain followed by a painful prolonged penile erection shortly after initiation of atomoxetine in a young adult receiving aripiprazole 7.5 mg daily for 6 months for bipolar I disorder. The episode is clinically significant because ischemic priapism can cause cavernosal smooth muscle injury and permanent erectile dysfunction if not treated promptly [1, 2]. The close temporal relationship to atomoxetine initiation and the absence of recurrence after discontinuation support a medication‐related trigger, while also highlighting the complexity introduced by concomitant antipsychotic therapy. Using the Naranjo Adverse Drug Reaction Probability Scale, the score was 4 (possible; Table S1), which supports a temporal medication‐event relationship but not a definitive confirmed causal attribution.

Pharmacologically induced priapism is increasingly recognized as a leading etiology of ischemic presentations, and antipsychotics account for a substantial proportion of drug‐related cases [7–9]. Medication‐associated priapism is most often reported with psychotropic agents, particularly antipsychotics. The most widely supported mechanism for antipsychotic‐associated priapism is α1‐adrenergic antagonism within the corpora cavernosa, which reduces sympathetic‐mediated detumescence and can promote veno‐occlusion [3, 7, 9]. However, α1 affinity alone does not fully predict clinical risk, suggesting contributions from additional pharmacologic pathways (e. g., serotonergic and dopaminergic effects that modulate autonomic tone or nitric‐oxide/cyclic guanosine monophosphate signaling), patient factors, and polypharmacy [3, 7, 9, 10].

Atomoxetine carries an FDA warning for priapism and has been associated with reports in children, adolescents, and adults. An FDA Drug Safety Communication (2013) notes that priapism appears to be reported more often with atomoxetine than with methylphenidate products, although the true incidence is unknown [6]. Published combination case reports further suggest vulnerability when atomoxetine is used with antipsychotics: Goetz and Surman [11] described prolonged penile erections in an 11‐year‐old receiving atomoxetine plus aripiprazole, and Bulut and Tanir [12] reported priapism after atomoxetine was added to risperidone in a child with autism spectrum disorder and ADHD. More recently, an adult Dutch case report described painful intermittent priapism that resolved after atomoxetine discontinuation, extending the association beyond pediatric populations [13]. A recent psychotropic‐priapism review likewise emphasizes that combination exposures and co‐prescribed vasoactive agents can complicate attribution and may increase risk [10].

Aripiprazole‐specific literature also warrants consideration in this case. Published case reports now include priapism after a single 10 mg oral dose, spontaneous‐resolution priapism in an adult with bipolar I disorder, and a pediatric case after inadvertent re‐exposure [14–16]. Together, these reports show that aripiprazole alone can precipitate painful prolonged erections despite its relatively low α1‐adrenergic receptor affinity. Overall, the present episode overlaps with both atomoxetine‐ and aripiprazole‐associated reports in featuring a painful erection occurring soon after medication initiation or change [11–17]. In the present case, onset occurred 5 days after starting atomoxetine, with detumescence approximately 6 h after symptom onset without intervention and no recurrence after discontinuation. Bilateral testicular pain is not commonly emphasized in published reports of atomoxetine‐associated priapism, though testicular pain is listed among adverse reactions in the U.S. prescribing information for atomoxetine [5], suggesting that this symptom may be underreported. The novelty of this case report is therefore not a first‐ever atomoxetine‐associated priapism signal, but a refined adult clinical characterization in the setting of stable aripiprazole co‐therapy and accompanying bilateral testicular pain.

The mechanism of atomoxetine‐associated priapism remains unclear. Atomoxetine inhibits the presynaptic norepinephrine transporter that increases synaptic norepinephrine (and indirectly increases dopamine in the prefrontal cortex) [5]. Although increased adrenergic tone might be expected to facilitate detumescence, medication‐associated priapism has been hypothesized to involve dysregulation of cavernosal smooth‐muscle tone, resulting in impaired venous outflow and subsequent corporal hypoxia and ischemia in susceptible individuals [1, 2, 10]. In addition, atomoxetine exposure is substantially higher in cytochrome P450 2D6 (CYP2D6) poor metabolizers and in the presence of strong CYP2D6 inhibitors; elevated exposure could plausibly increase the likelihood of rare adverse events [5]. Aripiprazole is metabolized primarily by dehydrogenation, hydroxylation, and N‐dealkylation. While dehydrogenation and hydroxylation are done by both CYP2D6 and CYP3A4, N‐dealkylation is catalyzed by 3A4. Clinically meaningful increases in aripiprazole exposure occur with strong inhibitors of either enzymes [18]. Cytochrome P450 2D6 also contributes to the metabolism of several psychotropics, including risperidone [19]; therefore, shared CYP2D6 metabolism is a pharmacokinetic consideration when these agents are coadministered, even though atomoxetine is not considered a clinically important CYP2D6 inhibitor based on available interaction data and prescribing information [5, 18, 19]. CYP2D6 genotype or metabolizer status was not assessed in this case; however, pharmacogenetic testing may be helpful in similar future cases when altered drug exposure is suspected. Accordingly, careful review of concomitant medications (including CYP2D6 and CYP3A4 inhibitors), OTC agents, supplements, and recreational substances is important when evaluating suspected medication‐associated priapism.

Concomitant aripiprazole remains an important alternative or contributing explanation. Although aripiprazole is often described as having relatively low α1‐adrenergic receptor affinity compared with some other second‐generation antipsychotics, it still exhibits measurable α1 binding and now has multiple published priapism case reports across adult and pediatric populations [3, 7, 9, 10, 14–16]. Signal detection analyses emphasize that priapism can occur with antipsychotics across a range of α1 affinities and that receptor affinity does not perfectly predict clinical risk [3]. In this patient, the stable aripiprazole regimen without prior events makes atomoxetine a plausible proximate trigger, but an aripiprazole contribution or an additive pharmacologic effect cannot be excluded.

Timing is variable and clinically relevant. Priapism has been reported shortly after initiation of ADHD medications, after dose increases, and during withdrawal or re‐challenge [6, 10–13, 17]; for antipsychotics, episodes may occur at any time during treatment and do not appear reliably dose‐dependent [3, 7, 9, 10, 14–16]. In this case, onset within days of starting atomoxetine, after a stable period on aripiprazole without priapism, supports atomoxetine as the more proximate trigger, but does not exclude an aripiprazole contribution or a combined pharmacologic effect.

The accompanying bilateral testicular pain may represent a related autonomic/vascular adverse effect. In this case, bilateral testicular pain preceded recognition of the prolonged erection and may therefore represent an early accompanying symptom that should prompt clinicians to inquire specifically about priapism in patients receiving atomoxetine. Testicular pain has been listed among reported adverse reactions in the U.S. prescribing information for atomoxetine, but published descriptions remain sparse [5]. Regardless of the mechanism, acute testicular pain concurrent with priapism should prompt urgent evaluation.

This report has limitations. Because cavernosal blood gas analysis and penile Doppler ultrasonography were not obtained, the episode is most appropriately described as suspected ischemic priapism based on the painful, rigid presentation, and clinical course rather than definitive vascular confirmation. We also did not perform re‐challenge (ethically inappropriate given the potential harm), and follow‐up duration was limited. The Naranjo scale was developed for single‐drug adverse reactions and does not reliably account for drug–drug interactions or pharmacodynamic additivity [20]. A Naranjo score of 4 indicates a possible adverse drug reaction; accordingly, the temporal association in this case is clinically suggestive but not confirmatory, particularly given concomitant aripiprazole exposure and the absence of rechallenge.

As a single case report without pharmacogenetic testing, objective vascular confirmation, serum drug‐level assessment, or rechallenge, this observation should be interpreted as hypothesis‐generating and not generalized beyond a plausible safety signal. Despite these constraints, this case adds to a small but growing body of literature describing priapism associated with atomoxetine alone, aripiprazole alone, and atomoxetine‐antipsychotic combinations, and it underscores the importance of comprehensive medication and substance review, patient counseling on warning signs, and prompt emergency evaluation when symptoms occur.

## 4. Conclusion

Priapism is rare, but prolonged ischemic episodes can lead to irreversible corporal injury and permanent erectile dysfunction [1, 2]. This case highlights painful prolonged erection with concurrent bilateral testicular pain occurring shortly after atomoxetine initiation in a patient receiving stable aripiprazole for bipolar I disorder.

The temporal association with atomoxetine initiation (onset 5 days after starting therapy) and the absence of recurrence after discontinuation make atomoxetine a plausible primary trigger; however, aripiprazole‐related priapism and/ or additive pharmacologic effects cannot be excluded [3, 7, 9, 10, 14–16]. Clinicians should obtain a comprehensive medication and substance history (including erectogenic agents and cytochrome P450 CYP2D6 and CYP3A4 inhibitors, counsel patients starting atomoxetine or antipsychotics about priapism, and advise immediate emergency evaluation for erections lasting ≥4 h [1–3, 10]. Clinicians should also be aware that priapism risk may differ across populations because inherited hematologic disorders linked to priapism, particularly sickle cell disease, are more prevalent in individuals of African, Afro‐Caribbean, and some Mediterranean ancestries. In addition, other inherited conditions, such as glucose‐6‐phosphate dehydrogenase deficiency, show population‐based variation, and racial/ethnic differences in cytochrome P450 enzymatic activity may influence psychotropic drug metabolism and vulnerability to adverse effects. Future ADHD treatment for this patient should also avoid re‐challenge with atomoxetine and incorporate shared decision‐making about alternative options (e. g., stimulants or α2‐agonists) with explicit counseling that priapism has also been reported rarely with stimulant medications and during dose changes or withdrawal [6, 17]. From a psychopharmacovigilance perspective, the case remains clinically relevant because rare, time‐sensitive adverse effects may first be recognized through detailed individual case reports, especially during psychotropic co‐therapy.

### 4.1. Learning Points

• Priapism is a rare urologic emergency; prolonged ischemic episodes can result in irreversible corporal injury and permanent erectile dysfunction, and erections lasting ≥4 h require immediate emergency evaluation [1, 2].
• Atomoxetine and antipsychotics have each been associated with priapism, and combined psychotropic regimens may increase vulnerability; a complete review of prescription and OTC medications, supplements, recreational substances, and cytochrome P450 interactions is essential [3, 5, 7, 9–19].
• Patients started on atomoxetine (especially when concurrently receiving antipsychotics) should be counseled about priapism and instructed to discontinue the suspected agent and seek urgent care if symptoms occur [3, 5, 7, 10–13, 17].

## Funding

No funding was received for this work.

## Ethics Statement

Institutional Review Board approval was not required for this single‐anonymized case report.

## Consent

Written informed consent for the publication of this case report, including the patient perspective, was obtained from the patient. The report was prepared in accordance with the CARE guidelines.

## Conflicts of Interest

The authors declare no conflicts of interest.

## Patient Perspective

When I started the new ADHD medicine, I did not expect to have pain in that area. I was worried that it might be something serious. I am glad it went away on its own, but I was told to get help quickly if it happens again. I feel more aware of what to look out for now.

## Supporting Information

Additional supporting information can be found online in the Supporting Information section.

## Supporting information


**Supporting Information** Completed Naranjo Adverse Drug Reaction Probability Scale for the reported case, supporting the causality assessment of the suspected adverse drug reaction. Naranjo Adverse Drug Reaction Probability Scale scoring for atomoxetine in this case.

## Data Availability

The data that support the findings of this study are available from the corresponding author upon reasonable request.
