# Supplementary material for: Atomoxetine‐Associated Bilateral Testicular Pain and Priapism in a Patient Taking Aripiprazole: A Case Report
Source: Case Rep Psychiatry. 2026 Jul 17;2026:6645625. doi: 10.1155/crps/6645625 (PMC13377404; doi:10.1155/crps/6645625)
Supplement: Supplementary file 1 — Supporting Information Completed Naranjo Adverse Drug Reaction Probability Scale for the reported case, supporting the causality assessment of the suspected adverse drug reaction. Naranjo Adverse Drug Reaction Probability Scale scoring for atomoxetine in this case. [file CRPS-2026-6645625-s001.docx]

**Supplementary Material**

Supplementary Table S1. Naranjo Adverse Drug Reaction Probability Scale scoring for atomoxetine in this case.

| Naranjo item | Response | Score | Rationale (case-specific) |
| --- | --- | --- | --- |
| 1. Are there previous conclusive reports on this reaction? | Yes | +1 | Priapism is listed in atomoxetine labeling and described in case reports/post-marketing reports. |
| 2. Did the adverse event appear after the suspected drug was administered? | Yes | +2 | Symptoms began 5 days after atomoxetine initiation. |
| 3. Did the adverse reaction improve when the drug was discontinued or a specific antagonist was administered? | Do not know | 0 | Detumescence occurred spontaneously before atomoxetine discontinuation. |
| 4. Did the adverse reaction reappear when the drug was readministered? | Not done | 0 | Re-challenge was not performed. |
| 5. Are there alternative causes that could on their own have caused the reaction? | Do not know | 0 | Concomitant aripiprazole and idiopathic priapism are possible but not confirmed. |
| 6. Did the reaction reappear when a placebo was given? | Not applicable | 0 | Not performed. |
| 7. Was the drug detected in blood (or other fluids) in concentrations known to be toxic? | Not done | 0 | No drug concentrations obtained. |
| 8. Was the reaction more severe when the dose was increased or less severe when the dose was decreased? | Not applicable | 0 | No dose changes occurred before the event. |
| 9. Did the patient have a similar reaction to the same or similar drugs in any previous exposure? | No/unknown | 0 | No prior history of priapism reported. |
| 10. Was the adverse event confirmed by any objective evidence? | Yes | +1 | Erection persisted >4 hours and was evaluated in the emergency department. |

Total Naranjo score: 4 (Possible adverse drug reaction).

Definite (≥9), Probable (5-8), Possible (1-4), Doubtful (≤0)

Note: The Naranjo scale was developed for single-drug adverse reactions and may not fully account for drug–drug interactions or pharmacodynamic additivity.
